# The relation between efavirenz versus nevirapine and virologic failure in Johannesburg, South Africa

**DOI:** 10.7448/IAS.17.1.19065

**Published:** 2014-10-22

**Authors:** Kate Shearer, Alana T Brennan, Mhairi Maskew, Lawrence Long, Rebecca Berhanu, Ian Sanne, Matthew P Fox

**Affiliations:** 1 Health Economics and Epidemiology Research Office, Department of Internal Medicine, School of Clinical Medicine, Faculty of Health Sciences, University of the Witwatersrand, Johannesburg, South Africa; 2 Center for Global Health & Development, Boston University, Boston, MA, USA; 3 Right to Care, Johannesburg, South Africa; 4 Clinical HIV Research Unit, Department of Internal Medicine, School of Clinical Medicine, Faculty of Health Sciences, University of the Witwatersrand, Johannesburg, South Africa; 5 Department of Epidemiology, School of Public Health, Boston University, Boston, MA, USA

**Keywords:** nevirapine, efavirenz, virologic failure, viral suppression, mortality, loss to follow-up, resource-limited settings

## Abstract

**Introduction:**

Previous research has raised concerns that patients given nevirapine (NVP)-based regimens experience more virologic failure than patients given efavirenz (EFV)-based regimens. We investigated this hypothesis in a cohort of HIV-positive patients at a large HIV treatment clinic in South Africa.

**Methods:**

All antiretroviral therapy (ART)-naïve non-pregnant patients, ≥18 years old, without tuberculosis, who initiated treatment with either NVP or EFV from April 2004 to August 2011 at the Themba Lethu Clinic in Johannesburg, South Africa, were included. Log-binomial regression and modified Poisson regression were used to estimate risk ratios (RR) with 95% confidence intervals (CI) for predictors of virologic failure, virologic suppression, and loss to follow-up (LTF), whereas a Cox proportional hazards model was used to estimate the risk of death, all within one year.

**Results:**

Of 12,840 included patients, 62.0% were female and the median baseline CD4 count was 98 cells/mm^3^ (36–169). Of these patients, 93.2% initiated an EFV-based regimen. After adjusting for baseline characteristics, no difference in death (adjusted Hazards Ratio (aHR): 0.92; 95% CI: 0.68–1.25), LTF (adjusted Risk Ratio (aRR): 1.00; 95% CI: 0.79–1.25), nor suppression (aRR: 0.98; 95% CI: 0.95–1.00) at one year was found between regimens. Among patients with ≥1 viral load ≥4 months after ART initiation, 4% (*n*=350) experienced virologic failure within 12 months of initiation. Patients initiating NVP-based regimens were 60% more likely to fail than patients initiating EFV-based regimens (aRR: 1.58; 95% CI: 1.13–2.22).

**Conclusions:**

In this cohort, patients initiating NVP-based regimens experienced more virologic failure than patients initiating EFV-based regimens. Future guidelines should consider the implications of different efficacy profiles when making recommendations for which drugs to prioritize.

## Introduction

The debate over which non-nucleoside reverse transcriptase inhibitor (NNRTI) should be prescribed in combination with antiretroviral therapy (ART) for the treatment of HIV infection has been growing recently. Under current guidelines released in June 2013, the World Health Organization (WHO) recommends efavirenz (EFV) with either lamivudine or emtricitabine and tenofovir for newly initiating adult patients [1]. Although updated in the 2013 guidelines, until recently in South Africa nevirapine (NVP) was recommended for pregnant women because of possible teratogenic effects of EFV, whereas EFV was recommended for patients co-infected with tuberculosis [2, 3]. The South African national treatment guidelines closely follow the recommendations of the WHO and caution the use of NVP for patients with higher CD4 counts because of the potential for severe adverse events [1–4]. As NVP is the less expensive drug, until recently, many national treatment programmes in Sub-Saharan Africa were still recommending its use [5–8] . While most countries are now recommending efavirenz over nevirapine, nevirapine is still commonly used. If outcomes on NVP-based regimens are inferior to that on EFV-based regimens, given the size of treatment programmes in Sub-Saharan Africa, this could lead to a substantial number of unnecessary treatment failures, regimen switches, and potentially, deaths.

Recent studies have indeed begun to fuel these concerns. A recent analysis combining cohorts from across the United States and Europe comparing NVP- to EFV-based regimens reported an increased risk of death and virologic failure among those taking NVP [9]. Likewise, a study from the United States also found an increased risk of virologic failure for NVP patients compared to EFV patients, and a recent review of all four WHO-recommended tenofovir regimens found an increased risk of virologic failure associated with a regimen of tenofovir–lamivudine–NVP compared to all others [10, 11]. A study from within the Southern African region using data prior to the large scale rollout of public-sector ART programmes also found an increased risk of virologic failure for patients prescribed NVP compared to EFV [12].

Although these studies provide some evidence of increased risk of virologic failure associated with NVP, few studies have been conducted to determine if this hypothesis holds in the public sector of resource-limited settings after the rollout of ART. Furthermore, most of these studies are subject to confounding by indication, as patients given NVP may be prescribed this drug for reasons which would be correlated with different overall health status than those prescribed EFV (e.g. pregnancy or a desire to get pregnant). In a recent publication, we examined this hypothesis amongst patients initiating ART with tenofovir and lamivudine and found an increased risk of failure for patients prescribed NVP over EFV [13]. In order to further investigate this hypothesis, we conducted a larger cohort analysis to assess the association between the initiating NNRTI and death, loss to follow-up (LTF), virologic suppression and virologic failure, in a cohort of ART-naïve adult patients in Johannesburg, South Africa, initiating standard first-line ART. We further used multidimensional bias analysis to explore whether any associations found were likely explained by unmeasured confounding.

## Methods

### Study site

The study was conducted at the Themba Lethu Clinic in Johannesburg, South Africa. Themba Lethu has been providing outpatient HIV care and treatment since 2004 when it opened as a public-sector clinic with NGO support. Patients are currently treated under the 2013 South African National Treatment guidelines which call for ART initiation at a CD4 count ≤350 cells/mm^3^or a WHO Stage III or IV condition [2].

Since Themba Lethu began initiating patients onto ART in 2004, more than 30,000 patients have received care and more than 12,000 patients are currently on treatment [14]. Under current guidelines, standard public-sector regimens for newly initiating patients in South Africa include tenofovir with either lamivudine or emtricitabine. Prior to the release of the 2010 guidelines, stavudine was the nucleoside reverse transcriptase inhibitor (NRTI) of choice in combination with ART in the public sector [15]. With the release of the 2013 guidelines, preference is given to EFV to complete standard triple-drug therapy, except in patients with psychiatric illness or shift workers [2]. During the period of this analysis, NVP was cautioned for women with CD4 counts >200/mm^3^ and men with CD4 counts >400 cells/mm^3^ [3].

All patient-level data, including information on demographics, medications, laboratory test results, clinical conditions, and other clinical details, are captured at the time of the patient encounter by a physician in an electronic medical record called TherapyEdge-HIV™ [14]. Routine laboratory tests (haemoglobin (Hb), alanine transaminase and creatinine) are conducted at the time of ART initiation. CD4 count is assessed at ART initiation and viral load is assessed four to six months post-ART initiation for treatment monitoring. Prior to 2010, CD4 and viral load were repeated every six months, but changes to the April 2010 guidelines called for these labs to be measured yearly thereafter. If a viral load result is >1000 copies/ml, a repeat viral load is conducted three months later.

For the first six months after initiating treatment patients are seen monthly. Once stable, patients are seen every two months for either a medical visit or for a medication pick up. Deaths are ascertained through passive follow-up and, for the 61% of patients who provide one, by linking national identification numbers with the National Vital Registration Infrastructure Initiative for patients who are LTF [16]. The linkage was last conducted in April 2013.

### Study population

We included all ART-naïve, adult (≥18 years old) patients who initiated ART between April 2004 and August 2011 at the Themba Lethu Clinic. We excluded patients who were either pregnant or co-infected with tuberculosis at the time of ART initiation as, during the period of this analysis, these patients should have received a specific regimen for cause. We additionally limited our analysis to patients who initiated onto a standard ART regimen under the guidelines. This meant lamivudine with a choice of tenofovir, zidovudine, or stavudine, and either NVP or EFV. Because of the recommendation that patients with high CD4 counts should not be prescribed NVP, we excluded women initiating NVP with a baseline CD4 count >200 cells/mm^3^, men initiating NVP with a baseline CD4 count >400 cells/mm^3^ and any patient without a baseline CD4 count from the analysis.

### Study variables

As the rollout of ART began in April of 2004, we defined the year of ART initiation in cohort years with each year starting in April and ending in March of the following year. Viral load and CD4 count results are uploaded directly into TherapyEdge-HIV™ from the South African National Health Laboratory Service (NHLS) on a daily basis. Baseline CD4 count was defined as the CD4 count taken closest to the date of ART initiation from six months prior to two weeks after the date of initiation, whereas the values for other baseline variables, namely body mass index (BMI) and Hb, were defined as the value closest to the data of ART initiation from 30 days prior to 7 days after ART initiation.

Baseline CD4 count was categorized as <50 cells/mm^3^, 50–99 cells/mm^3^, 100–199 cells/mm^3^ and ≥200 cells/mm^3^. For defining anaemia, Hb was adjusted downward by 0.65 g/dL to account for elevation above sea level in Johannesburg [17]. Anaemia was then categorized according to WHO guidelines as none (Hb ≥12 g/dL for non-pregnant women and Hb ≥13 g/dL for men), mild (Hb 11–11.9 g/dL for non-pregnant women and 11–12.9 g/dL for men), moderate (8–10.9 g/dL) and severe (Hb <8 g/dL). WHO Stage was determined either by physician classification or, in the absence of a physician's classification, by conditions present at ART initiation. BMI was categorized using standard cut-offs: <18.5, 18.5–24.9, 25–29.9, and ≥30.

### Statistical analysis

Demographic and clinical characteristics at baseline are presented using proportions for categorical variables and medians with corresponding interquartile ranges (IQR) for continuous variables. Our outcomes of interest were virologic failure, virologic suppression, death and LTF, all within 12 months of ART initiation. Virologic failure was defined as two consecutive (between two weeks and six months apart) viral loads >1000 copies/ml at least four months after ART initiation whereas virologic suppression was defined as one viral load <400 copies/ml. LTF was defined as being ≥3 months late for a scheduled visit with no subsequent visit.

For each outcome of interest, we present the proportion of the population experiencing the outcome. To determine predictors of virologic failure, virologic suppression, and LTF we used either log-binomial regression or modified Poisson regression with robust error estimation to estimate risk ratios (RR) and present them along with 95% confidence intervals (CI). We also present a Kaplan–Meier curve for the probability of death by drug regimen and hazard ratios for the risk of death from a Cox proportional hazards model. Age, sex, and baseline CD4 count were included in each adjusted model and other potential confounding variables that had a *p*<0.2 in univariate analyses were also included. All analyses were conducted using SAS version 9.3 (SAS Institute, Cary, North Carolina, USA).

### Sensitivity analyses

In order to determine whether levels of virologic failure remained elevated for NVP patients using different definitions of failure, we conducted a sensitivity analysis. For this analysis, we defined virologic failure at one year as: 1) two consecutive (between two weeks and six months apart) viral loads >400 copies/ml at least four months after ART initiation, 2) a single viral load >1000 copies/ml at least four months after treatment initiation and 3) either two consecutive failing viral loads >1000 copies/ml (between two weeks and six months apart) or one viral load >1000 copies/ml with no further documentation of suppression.

We conducted a second sensitivity analysis to see if the association between NVP and virologic failure remained when allowing for failure ever on treatment. We used our original definition of failure and included all patients with ≥1 viral load after four months on treatment.

### Bias analysis

In order to show the effect of a potential unmeasured confounder on the association between the initiating NNRTI and virologic failure, we also present the results of a multidimensional bias analysis that adjusts the analysis for such an unmeasured confounder. Each cell represents a measure of association of choice of NNRTI on virologic failure adjusted for the unmeasured confounder using assumptions about the prevalence of the unmeasured confounder among those given NVP and those given EFV, the strength of the association between the unmeasured confounder and virologic failure and standard formulas for correcting the unmeasured confounder [18, 19]. The analysis was carried out using freely available software (https://sites.google.com/site/biasanalysis/).

### Ethical approval

This analysis was approved by the Human Research Ethics Committee (Medical) of the University of the Witwatersrand and analysis of anonymized data was approved by the Institutional Review Board of Boston University.

## Results

### Demographic and clinical characteristics

Between April 2004 and August 2011, 15,850 adult patients initiated one of the regimens of interest at the Themba Lethu Clinic in Johannesburg, South Africa. We excluded 161 pregnant women, 2286 patients co-infected with tuberculosis, 111 patients initiated on NVP with high CD4 counts and 452 patients without a baseline CD4 count, leaving 12,840 patients in the final cohort for analysis.

Of the 12,840 who were included, 878 (6.8%) initiated a NVP-based regimen while 11,962 (93.2%) initiated an EFV-based regimen. Among the 878 who initiated a NVP-based regimen, most also initiated stavudine (82.9%) while the remainder initiated tenofovir (14.4%) and zidovudine (2.7%). Of those 11,962 patients who initiated an EFV-based regimen, a similar pattern was observed (77.2, 20.4, and 2.5% initiated stavudine, tenofovir, and zidovudine, respectively).

Among included patients, 62.0% were female, the median IQR age at ART initiation was 36.9 years (31.6–43.5) and the median IQR baseline CD4 count was 98 cells/mm^3^ (36–169). Although median CD4 counts at ART initiation were nearly identical across regimens (NVP: 100.5 cells/mm^3^; EFV: 98 cells/mm^3^), patients initiating EFV-based regimens were slightly older (median: 37.2 vs. 33.0), were more likely to be WHO stage III or IV (46.1% vs. 37.8%) and were somewhat more likely to have moderate or severe anaemia (48.3% vs. 43.3%) than patients who initiated a NVP-based regimen (Table 1).

**Table 1:** Patient characteristics at ART initiation and treatment outcomes for patients initiating a nevirapine- or efavirenz-based regimen between April 2004 and August 2011 in Johannesburg, South Africa

Variable	Exposure	Nevirapine	Efavirenz
Total *N*		878 (100%)	11,962 (100%)
Year of ART initiation^a^	2004/2005	227 (25.9%)	2513 (21.0%)
	2006/2007	266 (30.3%)	3247 (27.1%)
	2008/2009	242 (27.6%)	3485 (29.1%)
	2010/2011	143 (16.3%)	2717 (22.7%)
Sex	Male	312 (35.5%)	4566 (38.2%)
	Female	566 (64.5%)	7396 (61.8%)
Age (years)	Median (IQR)	33.0 (28.4–39.1)	37.2 (31.9–43.9)
	<30	283 (32.2%)	2033 (17.0%)
	30–34	243 (27.7%)	2672 (22.3%)
	35–39	169 (19.3%)	2644 (22.1%)
	40–44	103 (11.7%)	1963 (16.4%)
	≥45	80 (9.1%)	2650 (22.2%)
CD4 count (cells/mm^3^)	Median (IQR)	100.5 (42–160)	98 (36–169)
	<50	242 (27.6%)	3741 (31.3%)
	50–99	192 (21.9%)	2305 (19.3%)
	100–199	394 (44.9%)	4181 (35.0%)
	≥200	50 (5.7%)	1735 (14.5%)
WHO Stage	Stage I	419 (47.7%)	4519 (37.8%)
	Stage II	127 (14.5%)	1932 (16.2%)
	Stage III	247 (28.1%)	3585 (30.0%)
	Stage IV	85 (9.7%)	1926 (16.1%)
BMI (kg/m^2^)	Median (IQR)	22.0 (19.7–25.1)	21.7 (19.2–24.9)
	Missing	75 (8.5%)	1169 (9.8%)
	<18.5	114 (13.0%)	2034 (17.0%)
	18.5–24.9	481 (54.8%)	6125 (51.2%)
	25.0–29.9	136 (15.5%)	1823 (15.2%)
	≥30	72 (8.2%)	811 (6.8%)
Haemoglobin (g/dL)	Median (IQR)	11.3 (9.7–12.7)	11.0 (9.5–12.5)
Anaemia	Missing	50 (5.7%)	569 (4.8%)
	No anaemia	236 (26.9%)	2900 (24.2%)
	Mild anaemia	212 (24.2%)	2720 (22.7%)
	Moderate anaemia	317 (36.1%)	4627 (38.7%)
	Severe anaemia	63 (7.2%)	1146 (9.6%)
NRTI^b^	Stavudine	728 (82.9%)	9235 (77.2%)
	Zidovudine	24 (2.7%)	293 (2.5%)
	Tenofovir	126 (14.4%)	2434 (20.4%)
Outcome at 12 months	Alive	700 (79.7%)	9158 (76.6%)
	Dead	55 (6.3%)	1006 (8.4%)
	Lost to follow-up	88 (10.0%)	1287 (10.8%)
	Transferred out	35 (4.0%)	511 (4.3%)
Achieved viral suppression^c^		641 (90.0%)	8571 (92.7%)
Experienced virologic failure^d^		43 (6.1%)	307 (3.3%)

a A year is defined as a cohort year, starting 1 April and ending 31 March of the following year. The 2011 cohort starts in April and ends in August of the same year;

b nucleoside reverse transcriptase inhibitor (NRTI);

c among patients with at least one viral load between one month and one year after ART initiation;

d among patients with at least one viral load after four months of follow-up.

### Death and LTF

**Figure 1:**
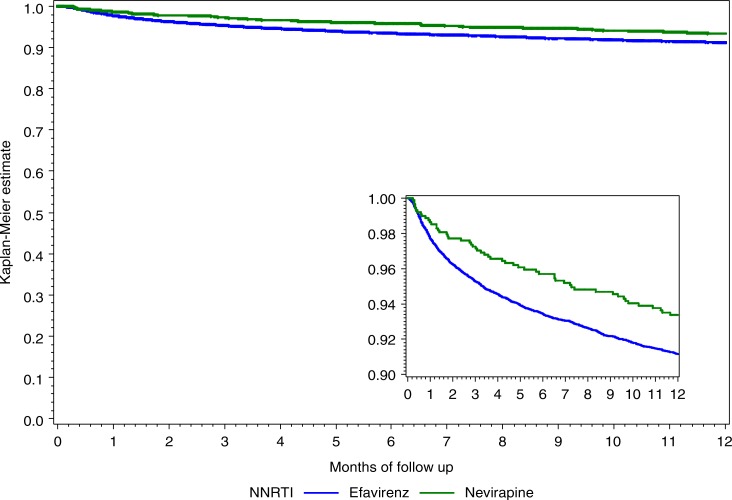
Kaplan–Meier Curve for time to death according to baseline NNRTI among ART-naïve, adult patients initiating either a nevirapine- or efavirenz-based regimen between April 2004 and August 2011 in Johannesburg, South Africa.

Of the 12,840 patients included in the analysis, 1061 patients died (8.3%) within the first 12 months on ART, with EFV patients slightly more likely to die than NVP patients (8.4 vs. 6.3%) (Figure 1). Patients died in a median IQR of 2.6 (1.0–5.8) months on treatment. When adjusted for baseline characteristics including sex, age and CD4 count, we did not identify any difference in death according to regimen (adjusted Hazards Ratio (aHR) NVP versus EFV: 0.92; 95% CI: 0.68–1.25) (Table 2).

**Table 2:** Unadjusted and adjusted predictors of death and loss to follow-up among patients initiated on a nevirapine- or efavirenz-based regimen between April 2004 and August 2011 in Johannesburg, South Africa

	Death within 12 months (*n*=12,840)			Loss to follow-up within 12 months (*n*=12,059)		
		
Characteristic	Death/N (%)	Unadjusted HR (95% CI)	Adjusted HR (95% CI)	LTF/N (%)	Unadjusted RR (95% CI)	Adjusted RR (95% CI)
Regimen						
** **Nevirapine^a^	55/878 (6.3%)	0.73 (0.56, 0.96)	0.92 (0.68, 1.25)	88/843 (10.4%)	0.91 (0.74, 1.12)	1.00 (0.79, 1.25)
** **Efavirenz^b^	1006/11,962 (8.4%)	*Reference*	*Reference*	1287/11,216 (11.5%)	*Reference*	*Reference*
Year of ART initiation						
** **2004/2005	220/2740 (8.0%)	*Reference*	*Reference*	247/2623 (9.4%)	*Reference*	*Reference*
** **2006/2007	346/3513 (9.9%)	1.26 (1.07, 1.50)	1.22 (1.01, 1.48)	379/3281 (11.6%)	1.23 (1.05, 1.43)	1.19 (1.01, 1.41)
** **2008/2009	347/3727 (9.3%)	1.19 (1.01, 1.41)	1.24 (1.02, 1.49)	374/3463 (10.8%)	1.15 (0.98, 1.34)	1.07 (0.90, 1.28)
** **2010/2011	148/2860 (5.2%)	0.66 (0.54, 0.82)	0.63 (0.42, 0.95)	375/2692 (13.9%)	1.48 (1.27, 1.72)	1.78 (1.35, 2.34)
Sex						
** **Male	492/4878 (10.1%)	1.45 (1.29, 1.64)	1.41 (1.21, 1.64)	634/4527 (14.0%)	1.42 (1.29, 1.57)	1.38 (1.22, 1.56)
** **Female	569/7962 (7.2%)	*Reference*	*Reference*	741/7532 (9.8%)	*Reference*	*Reference*
Age at initiation						
** **<30	152/2316 (6.6%)	*Reference*	*Reference*	294/2184 (13.5%)	*Reference*	*Reference*
** **30–34	202/2915 (6.9%)	1.05 (0.85, 1.30)	0.98 (0.77, 1.25)	316/2750 (11.5%)	0.85 (0.74, 0.99)	0.86 (0.73, 1.02)
** **35–39	238/2813 (8.5%)	1.29 (1.05, 1.58)	1.29 (1.03, 1.63)	287/2641 (10.9%)	0.81 (0.69, 0.94)	0.74 (0.62, 0.89)
** **40–44	180/2066 (8.7%)	1.33 (1.07, 1.65)	1.48 (1.16, 1.88)	219/1949 (11.2%)	0.83 (0.71, 0.98)	0.78 (0.64, 0.94)
** **≥45	289/2730 (10.6%)	1.63 (1.34, 1.99)	1.84 (1.47, 2.31)	259/2535 (10.2%)	0.76 (0.65, 0.89)	0.71 (0.59, 0.85)
Baseline CD4+ Count (cells/mm^3^)						
** **<50	581/3983 (14.6%)	4.47 (3.45, 5.79)	3.03 (2.20, 4.17)	500/3560 (14.0%)	1.49 (1.26, 1.75)	1.13 (0.92, 1.39)
** **50–99	202/2497 (8.1%)	2.34 (1.76, 3.10)	2.07 (1.48, 2.90)	268/2359 (11.4%)	1.20 (1.00, 1.44)	1.05 (0.84, 1.31)
** **100–199	214/4575 (4.7%)	1.32 (1.00, 1.75)	1.39 (1.00, 1.94)	443/4406 (10.1%)	1.06 (0.90, 1.26)	1.12 (0.92, 1.37)
** **≥200	64/1785 (3.6%)	*Reference*	*Reference*	164/1734 (9.5%)	*Reference*	*Reference*
WHO Stage						
** **Stage I	256/4938 (5.2%)	*Reference*	*Reference*	482/4754 (10.1%)	*Reference*	*Reference*
** **Stage II	113/2059 (5.5%)	1.06 (0.85, 1.32)	1.10 (0.86, 1.39)	184/1980 (9.3%)	0.92 (0.78, 1.08)	0.94 (0.79, 1.12)
** **Stage III	381/3832 (9.9%)	1.99 (1.70, 2.33)	1.37 (1.14, 1.64)	401/3561 (11.3%)	1.11 (0.98, 1.26)	0.94 (0.81, 1.09)
** **Stage IV	311/2011 (15.5%)	3.32 (2.81, 3.92)	1.69 (1.38, 2.07)	308/1764 (17.5%)	1.72 (1.51, 1.97)	1.06 (0.89, 1.26)
Anaemia						
** **No anaemia	111/3136 (3.5%)	*Reference*	*Reference*	279/3055 (9.1%)	*Reference*	*Reference*
** **Mild anaemia	171/2932 (5.8%)	1.67 (1.31, 2.11)	1.34 (1.03, 1.74)	257/2823 (9.1%)	1.00 (0.85, 1.17)	0.92 (0.77, 1.09)
** **Moderate anaemia	520/4944 (10.5%)	3.15 (2.56, 3.86)	2.38 (1.88, 3.00)	552/4566 (12.1%)	1.32 (1.15, 1.52)	1.17 (1.00, 1.38)
** **Severe anaemia	204/1209 (16.9%)	5.53 (4.39, 6.97)	3.73 (2.83, 4.90)	204/1041 (19.6%)	2.15 (1.82, 2.53)	1.46 (1.18, 1.81)
BMI (kg/m^2^)						
** **<18.5	311/2148 (14.5%)	2.57 (2.22, 2.98)	1.69 (1.44, 1.97)	302/1924 (15.7%)	1.62 (1.42, 1.84)	1.36 (1.19, 1.56)
** **18.5–24.9	404/6606 (6.1%)	*Reference*	*Reference*	612/6302 (9.7%)	*Reference*	*Reference*
** **25–29.9	89/1959 (4.5%)	0.73 (0.58, 0.92)	0.92 (0.73, 1.18)	130/1897 (6.9%)	0.71 (0.59, 0.85)	0.80 (0.66, 0.96)
** **≥30	58/883 (6.6%)	1.04 (0.79, 1.38)	1.67 (1.25, 2.22)	45/858 (5.2%)	0.54 (0.40, 0.72)	0.67 (0.50, 0.91)
NRTI						
** **Stavudine	912/9963 (9.2%)	*Reference*	*Reference*	1026/9351 (11.0%)	*Reference*	*Reference*
** **Zidovudine	23/317 (7.3%)	0.81 (0.53, 1.22)	0.90 (0.55, 1.49)	41/294 (14.0%)	1.27 (0.95, 1.70)	1.34 (0.95, 1.88)
** **Tenofovir	126/2560 (4.9%)	0.54 (0.45, 0.65)	0.95 (0.64, 1.43)	308/2414 (12.8%)	1.16 (1.03, 1.31)	0.75 (0.58, 0.98)

a Nevirapine regimen includes tenofovir–lamivudine–nevirapine, zidovudine–lamivudine–nevirapine, stavudine–lamivudine–nevirapine;

b efavirenz regimen includes tenofovir–lamivudine–efavirenz, zidovudine–lamivudine–efavirenz, stavudine–lamivudine–efavirenz.

As a patient cannot be considered LTF before three months on treatment, patients with less than three months of follow-up were excluded from the analysis of loss, leaving 12,059 patients in the cohort. LTF was similar across treatment regimens (11.5% vs. 10.4% for EFV and NVP patients, respectively) and no difference in LTF was found after adjusting for baseline characteristics (adjusted Risk Ratio (aRR): 1.00; 95% CI: 0.79–1.25) (Table 2).

### Virologic suppression

Between one month and one year after ART initiation, 9960 (77.6%) patients had at least one viral load conducted. Among these patients, viral suppression was common (*n*=9212; 92.5%) with little variation between regimens both before (RR NVP vs. EFV: 0.97; 95% CI: 0.95–1.00) and after adjusting for baseline characteristics (aRR NVP vs. EFV: 0.98; 95% CI: 0.95–1.00) (Table 3).

**Table 3:** Unadjusted and adjusted predictors of virologic suppression and virologic failure among patients initiated on a nevirapine- or efavirenz-based regimen between April 2004 and August 2011 in Johannesburg, South Africa

	Suppression within 12 months (*n*=9960)			Failure within 12 months (*n*=8854)		
		
Characteristic	Suppression/*N* (%)	Unadjusted RR (95% CI)	Adjusted RR (95% CI)	Failures/*N*(%)	Unadjusted RR (95% CI)	Adjusted RR (95% CI)
Regimen						
** **Nevirapine^a^	641/712 (90.0%)	0.97 (0.95, 1.00)	0.98 (0.95, 1.00)	43/643 (6.7%)	1.79 (1.31, 2.44)	1.58 (1.13, 2.22)
** **Efavirenz^b^	8571/9248 (92.7%)	*Reference*	*Reference*	307/8211 (3.7%)	*Reference*	*Reference*
Year of ART initiation						
** **2004/2005	1981/2179 (90.9%)	*Reference*	*Reference*	72/1944 (3.7%)	*Reference*	*Reference*
** **2006/2007	2600/2718 (95.7%)	1.05 (1.04, 1.07)	1.05 (1.03, 1.06)	93/2405 (3.9%)	1.04 (0.77, 1.41)	1.06 (0.78, 1.45)
** **2008/2009	2756/2870 (96.0%)	1.06 (1.04, 1.07)	1.05 (1.03, 1.06)	42/2404 (1.8%)	0.47 (0.32, 0.69)	0.51 (0.35, 0.76)
** **2010/2011	1875/2193 (85.5%)	0.94 (0.92, 0.96)	0.94 (0.90, 0.98)	143/2101 (6.8%)	1.84 (1.39, 2.42)	1.96 (1.17, 3.28)
Sex						
** **Male	3266/3601 (90.7%)	0.97 (0.96, 0.98)	1.03 (1.02, 1.05)	139/3186 (4.4%)	1.17 (0.95, 1.45)	1.23 (0.98, 1.54)
** **Female	5946/6359 (93.5%)	*Reference*	*Reference*	211/5668 (3.7%)	*Reference*	*Reference*
Age at initiation						
** **<30	1616/1769 (91.4%)	*Reference*	*Reference*	80/1565 (5.1%)	*Reference*	*Reference*
** **30–34	2098/2281 (92.0%)	1.01 (0.99, 1.03)	1.01 (0.99, 1.03)	84/2045 (4.1%)	0.80 (0.60, 1.08)	0.84 (0.61, 1.14)
** **35–39	2061/2224 (92.7%)	1.01 (1.00, 1.03)	1.02 (1.00, 1.04)	74/1995 (3.7%)	0.73 (0.53, 0.99)	0.73 (0.53, 1.01)
** **40–44	1460/1589 (91.9%)	1.01 (0.99, 1.03)	1.01 (0.99, 1.03)	51/1390 (3.7%)	0.72 (0.51, 1.01)	0.69 (0.48, 0.99)
** **≥45	1977/2097 (94.3%)	1.03 (1.01, 1.05)	1.03 (1.01, 1.05)	61/1859 (3.3%)	0.64 (0.46, 0.89)	0.68 (0.48, 0.96)
Baseline CD4+ Count (cells/mm^3^)						
** **<50	2545/2796 (91.0%)	0.97 (0.95, 0.99)	0.96 (0.95, 0.98)	140/2480 (5.7%)	2.57 (1.73, 3.81)	2.74 (1.80, 4.17)
** **50–99	1784/1953 (91.4%)	0.97 (0.95, 0.99)	0.97 (0.95, 0.99)	72/1725 (4.2%)	1.90 (1.24, 2.91)	1.96 (1.25, 3.06)
** **100–199	3486/3724 (93.6%)	1.00 (0.98, 1.01)	0.99 (0.97, 1.01)	109/3328 (3.3%)	1.49 (1.00, 2.24)	1.52 (1.00, 2.31)
** **≥200	1397/1487 (94.0%)	*Reference*	*Reference*	29/1321 (2.2%)	*Reference*	*Reference*
WHO Stage						
** **Stage I	3726/4013 (92.9%)	*Reference*	*Reference*	127/3577 (3.6%)	*Reference*	–
** **Stage II	1584/1697 (93.3%)	1.01 (0.99, 1.02)	1.01 (0.99, 1.03)	55/1503 (3.7%)	1.03 (0.76, 1.41)	–
** **Stage III	2702/2932 (92.2%)	0.99 (0.98, 1.01)	1.00 (0.98, 1.01)	118/2607 (4.5%)	1.27 (1.00, 1.63)	–
** **Stage IV	1200/1318 (91.1%)	0.98 (0.96, 1.00)	1.00 (0.98, 1.02)	50/1167 (4.3%)	1.21 (0.88, 1.66)	–
Anaemia						
** **No anaemia	2452/2626 (93.4%)	*Reference*	*Reference*	74/2336 (3.2%)	*Reference*	*Reference*
** **Mild anaemia	2244/2421 (92.7%)	0.99 (0.98, 1.01)	1.00 (0.98, 1.01)	81/2136 (3.8%)	1.20 (0.88, 1.63)	1.10 (0.81, 1.50)
** **Moderate anaemia	3423/3717 (92.1%)	0.99 (0.97, 1.00)	0.99 (0.98, 1.01)	148/3310 (4.5%)	1.41 (1.07, 1.86)	1.24 (0.93, 1.65)
** **Severe anaemia	715/764 (93.6%)	1.00 (0.98, 1.02)	1.02 (1.00, 1.04)	26/675 (3.9%)	1.22 (0.78, 1.89)	1.03 (0.66, 1.62)
BMI						
** **<18.5	1348/1480 (91.1%)	0.98 (0.97, 1.00)	0.99 (0.97, 1.01)	62/1287 (4.8%)	1.28 (0.96, 1.69)	–
** **18.5–24.9	4948/5349 (92.5%)	*Reference*	*Reference*	180/4774 (3.8%)	*Reference*	–
** **25–29.9	1574/1678 (93.8%)	1.01 (1.00, 1.03)	1.01 (1.00, 1.03)	53/1515 (3.5%)	0.93 (0.69, 1.25)	–
** **≥30	730/763 (95.7%)	1.03 (1.02, 1.05)	1.02 (1.00, 1.04)	22/661 (3.3%)	0.88 (0.57, 1.36)	–
NRTI						
** **Stavudine	7276/7737 (94.0%)	*Reference*	*Reference*	221/6746 (3.3%)	*Reference*	*Reference*
** **Zidovudine	219/237 (92.4%)	0.98 (0.95, 1.02)	0.98 (0.94, 1.02)	3/213 (1.4%)	0.43 (0.14, 1.33)	0.49 (0.16, 1.51)
** **Tenofovir	1717/1986 (86.5%)	0.92 (0.90, 0.94)	1.00 (0.96, 1.04)	126/1895 (6.7%)	2.03 (1.64, 2.51)	1.14 (0.70, 1.85)

a Nevirapine regimen includes tenofovir–lamivudine–nevirapine, zidovudine–lamivudine–nevirapine, stavudine–lamivudine–nevirapine;

b efavirenz regimen includes tenofovir–lamivudine-efavirenz, zidovudine–lamivudine–efavirenz, stavudine–lamivudine–efavirenz.

### Virologic failure

Virologic failure occurred infrequently in the first year on treatment. Among the 8854 patients with at least one viral load between 4 and 12 months on treatment, only 4.0% (*n*=350) experienced virologic failure and 90.6% of those patients failed while on their initiating regimen. However, a higher proportion of patients prescribed NVP experienced failure in the first year on ART compared to patients initiated on an EFV-based regimen (6.7% vs. 3.7%).

In an unadjusted analysis, NVP patients were approximately 80% more likely to experience failure compared to EFV patients (RR: 1.79; 95% CI: 1.31–2.44). After adjusting for year of ART initiation, sex, age, baseline CD4 count, baseline anaemia and NRTI, a strong association remained and NVP patients were almost 60% more likely to fail than EFV patients (aRR: 1.58; 95% CI: 1.13–2.22) (Table 3).

In order to determine whether or not there was a difference in the association between NNRTI and virologic failure by sex, given possible exposure to single-dose NVP as part of prevention of mother-to-child transmission (PMTCT) programmes, we conducted an analysis stratified by sex. In an unadjusted model, male patients prescribed NVP were 32% more likely to fail compared to EFV patients (RR: 1.32; 95% CI: 0.74–2.34) whereas female patients prescribed NVP were more than twice as likely to fail as EFV patients (RR: 2.10; 95% CI: 1.45–3.03). However, when adjusted for year of ART initiation, age, baseline CD4 count, baseline anaemia level and NRTI, the difference between sexes was reduced. The RR of failure comparing NVP to EFV for males was 1.40 (95% CI: 0.77–2.55), whereas for women it was 1.64 (95% CI: 1.08–2.49).

### Sensitivity analyses

For the first sensitivity analysis, using the first definition of virologic failure requiring two failing viral loads >400 copies/ml, NVP patients were more than 50% more likely to fail (RR: 1.53; 95% CI: 1.15–2.04) compared to EFV patients, with a similar risk after adjusting for year of ART initiation, age, sex, baseline CD4 count, BMI and NRTI (aRR: 1.52; 95% CI: 1.11–2.08). With the second definition requiring just one single failing viral load >1000 copies/ml, NVP patients were still more likely to fail than EFV patients in both unadjusted (RR: 1.31; 95% CI: 1.12–1.54) and adjusted models (aRR: 1.39; 95% CI: 1.20–1.63). Requiring that the single failing viral load be conducted while still on the initiating regimen resulted in an adjusted RR of 1.27 (95% CI: 1.07–1.52) and requiring that there also be no evidence of further suppression on the original regimen resulted in an adjusted RR of 1.63 (95% CI: 1.27–2.09). Including only patients with at least six months of follow-up (*n*=10,851) and re-classifying patients with missing viral loads as virologic failure (*n*=2023; 18.6%) resulted in an adjusted RR of 1.09 (95% CI: 0.98–1.22). Using the final definition of either two failing viral loads or one failing viral load with no further evidence of suppression resulted in an adjusted RR of 1.59 (95% CI: 1.25–2.02).

A total of 9815 patients (9108 [92.8%] on EFV and 707 on NVP [7.2%]) had at least one viral load ever on treatment (but at least four months after initiation) and were included in the second sensitivity analysis. In this group, 18.5% of NVP patients and 11.6% of EFV patients failed (two consecutive failing viral loads >1000 copies/ml) ever on treatment resulting in an unadjusted RR of 1.60 (95% CI: 1.36–1.89). After adjustment for baseline characteristics, NVP patients were still almost 40% more likely to fail ever on treatment than EFV patients (aRR: 1.38; 95% CI: 1.15, 1.65).

### Bias analysis

Most patients in our database were prescribed an EFV-based regimen, as would be expected given the treatment guidelines. The most likely reason for being prescribed NVP is pregnancy or a desire to get pregnant, conditions that are not likely to be associated with greater illness severity or treatment failure. However, given NVP may be prescribed for a particular cause, it is possible confounding by indication explains some or all of the increased failure associated with NVP. For example, while we excluded patients with tuberculosis or women who were pregnant, it is possible our database was not complete and we did in fact include some of these patients in our analysis. In addition EFV is not recommended for patients with a history of mental illness; [4] however, history of mental illness is not recorded in our database. To account for such possible unmeasured confounding, we conducted a multidimensional bias analysis (see Appendix) for the potential effect of an unmeasured confounder on the association between NNRTI and virologic failure.

We found that in order for the true association between the initiating NNRTI and virologic failure to be null, the prevalence of the potential confounder would have to be very high among NVP patients and very low among EFV patients and have a strong association with virologic failure. For example, if 70% of NVP patients but only 13% of EFV patients had the unmeasured confounder and if the RR for the effect of the confounder on virologic failure was 2.69, adjustment for the confounder would result in no association between NVP use and virologic failure. As a confounder with such prevalence distributions between NVP and EFV that is also uncorrelated with any other variables we adjusted for is unlikely, while we may be overestimating the effect of NVP on virologic failure due to unmeasured confounding, it is unlikely that the true association is null.

## Discussion

In this cohort of ART-naïve adult patients, patients prescribed a NVP-based regimen were much more likely to experience virologic failure within the first 12 months on treatment compared to patients initiated onto an EFV-based regimen, even after adjustment for age, sex, baseline anaemia and baseline CD4 count. This relationship was found despite the fact that we attempted to exclude patients who would be prescribed a regimen for reasons related to treatment failure (i.e. those with tuberculosis and those pregnant at ART initiation).

Although studies have found conflicting results, [20, 21] our findings corroborate the evidence presented in several studies that patients initiated on NVP are at higher risk for virologic failure than patients initiated on EFV. In both the Italian Cohort Naïve Antiretrovirals study and an observational cohort analysis of more than 10,000 people, NVP patients were also twice as likely to fail compared to EFV patients [22, 23]. In their recent publication, the HIV Causal Collaboration determined that in their cohort of American and European patients, after adjusting for baseline characteristics, NVP patients were more than 50% more likely to fail than EFV patients (RR: 1.53; 95% CI: 1.40, 1.69) [9]. A similar result was found by Nachega *et al*. in their study of Southern African adults (HR: 1.52; 95% CI: 1.34, 2.02) [12]. A study from the United States published by van den Berg-Wolf *et al*. also found increased risk of failure on NVP (using a failure cut-off of 1000 copies/ml); however, the effect was less pronounced (aHR EFV vs. NVP: 0.80; 95% CI: 0.58, 1.10) [10].

Our study has several strengths. First, by removing pregnant women and patients co-infected with tuberculosis, we were able to reduce confounding by indication in this cohort. Second, as laboratory results are uploaded directly from the NHLS, we are able to reduce data entry errors and omissions. Third, as we link patients with South African national identification numbers to the National Vital Registration System, we are able to improve ascertainment of deaths in addition to ongoing passive surveillance.

This study, however, should also be viewed in light of several limitations. As we do not collect information on adherence, we were unable to control for its effect on virologic outcomes. Further, as PMTCT services are not provided at Themba Lethu, data on prior exposure to single-dose NVP or other PMTCT regimens is limited and we were unable to control for the effects of those exposures. The OCTANE study did find an increased risk of virologic failure for women previously exposed to single-dose NVP who were prescribed a NVP-based regimen compared to a ritonavir-boosted lopinavir-based regimen [24]. However, as we also found an effect in men in our stratified analysis, we believe the impact of PMTCT regimens is minimal in this cohort. In addition, as only 61% of patients provided a valid national identification number, [16] while linking with the National Vital Registration System does improve ascertainment of deaths, we may still be underestimating mortality in this cohort. Finally, as NVP is less often prescribed in this cohort than EFV, the sample size for NVP-based regimens was small and readers should use caution when interpreting the results.

## Conclusions

This analysis provides additional support to evidence provided in other studies that patients prescribed NVP-based regimens experience more virologic failure than patients prescribed EFV-based regimens. However, additional studies with longer follow-up are needed to determine if this increased risk of failure is common across all NVP-based regimens or if it is confined to specific nucleoside reverse transcriptase inhibitor backbones.

## Conference presentation

This analysis was presented as a poster at the 6th South African AIDS Conference in Durban, South Africa held from the 18th to the 21st of June 2013.

## Appendix

**Table:** Appendix. JacanaResults from the bias analysis to control for the effect of a potential unmeasured confounder on the association between initiating NNRTI and virologic failure among ART-naïve, adult patients who initiated a nevirapine- or efavirenz-based regimen between April 2004 and August 2011 in Johannesburg, South Africa

Multidimensional RR NVP-Failure Relationship Adjusted for Confounder												

		Risk Ratio: Confounder-Failure										
		
p(Confounder∣NVP)	p(Confounder∣EFV)	2.69	2.79	2.89	2.99	3.09	3.19	3.29	3.39	3.49	3.59	3.69
0.7	0.13	1.00	0.98	0.96	0.94	0.92	0.91	0.89	0.88	0.86	0.85	0.84
0.72	0.14	1.00	0.98	0.96	0.94	0.92	0.91	0.89	0.88	0.86	0.85	0.84
0.74	0.15	1.00	0.98	0.96	0.94	0.92	0.91	0.89	0.88	0.86	0.85	0.84
0.76	0.16	0.99	0.97	0.96	0.94	0.92	0.91	0.89	0.88	0.86	0.85	0.84
0.78	0.17	0.99	0.97	0.96	0.94	0.92	0.91	0.89	0.88	0.87	0.85	0.84
0.8	0.18	0.99	0.97	0.95	0.94	0.92	0.91	0.89	0.88	0.87	0.85	0.84
0.82	0.19	0.99	0.97	0.95	0.94	0.92	0.91	0.89	0.88	0.87	0.85	0.84
0.84	0.2	0.99	0.97	0.95	0.94	0.92	0.91	0.89	0.88	0.87	0.85	0.84
0.86	0.21	0.99	0.97	0.95	0.94	0.92	0.91	0.89	0.88	0.87	0.86	0.84
0.88	0.22	0.99	0.97	0.95	0.93	0.92	0.91	0.89	0.88	0.87	0.86	0.85
0.9	0.23	0.99	0.97	0.95	0.93	0.92	0.91	0.89	0.88	0.87	0.86	0.85
